# Plasma C‐reactive protein and interleukin‐6 concentrations in foals during health and respiratory disease

**DOI:** 10.1111/evj.70000

**Published:** 2025-07-20

**Authors:** Dorothea Hildebrandt, Monica Venner, Kelsey A. Hart, Londa Berghaus

**Affiliations:** ^1^ Clinic for Horses, University of Veterinary Medicine Hannover Hanover Germany; ^2^ Equine Clinic Destedt Germany; ^3^ Department of Large Animal Medicine University of Georgia Athens Georgia USA

**Keywords:** bronchopneumonia, C‐reactive protein, foals, horse, inflammation, interleukin‐6

## Abstract

**Background:**

Early and specific diagnosis of bronchopneumonia in foals is important to prevent severe disease. In human medicine, C‐reactive protein (CRP) and interleukin‐6 (IL‐6) are important diagnostic and prognostic biomarkers in neonatal pneumonia in other species. Evaluation of these markers in foals with naturally occurring respiratory diseases is lacking.

**Objectives:**

To determine if CRP and IL‐6 were useful predictors of respiratory disease in foals from birth to weaning.

**Study Design:**

Prospective cohort study.

**Methods:**

Periodic blood samples from 200 initially healthy foals were collected from birth to weaning on a farm with endemic *Rhodococcus equi* and *Streptococcus equi* pneumonia. The foals were examined weekly by physical examination and trans‐thoracic ultrasonography to determine the presence or absence of pulmonary consolidation and were divided into three groups after weaning: (1) foals that remained healthy; (2) foals that developed subclinical, mild, self‐limiting pulmonary lesions; and (3) foals that developed severe pulmonary lesions and clinical pneumonia that required antimicrobial treatment. Thirty foals from each health group (*N* = 90 total foals) were randomly selected from the 200 initially enrolled for assessment of associations between CRP and IL‐6 concentrations and health status. Data were analysed using linear mixed models, with *p*‐values < 0.05 considered statistically significant.

**Results:**

Age‐related changes were found in both plasma CRP and IL‐6 concentrations. Circulating concentrations of CRP were increased through weaning, while plasma IL‐6 concentrations decreased through weaning. Respiratory disease did not significantly impact concentrations of CRP or IL‐6 at any age.

**Main Limitations:**

Timing of sample collection, small sample size.

**Conclusions:**

Neither IL‐6 nor CRP concentrations were suitable predictors of subclinical or clinical bronchopneumonia in foals in this study. Further studies are needed to determine if more frequent measurement of these markers in foals at the time of pneumonia diagnosis provides helpful diagnostic or prognostic information.

## 1 INTRODUCTION

Pneumonia is one of the most important diseases in growing foals. *Rhodococcus equi* (*R. equi*) and *Streptococcus equi subsp. zooepidemicus* (*Strep. zoo*.) are known to be the most common causes of this disease in foals.^1^, ^2^, ^3^, ^4^ Early and specific diagnosis is essential, especially on endemic farms, to help make decisions for the appropriate treatment and prevent pneumonia‐associated morbidity and mortality. Several diagnostic methods have been evaluated for the diagnosis of pneumonia in foals, such as thoracic radiography, trans‐thoracic ultrasonography, and haematologic parameters such as leukocyte count, fibrinogen concentration, and serum amyloid A concentration.^5^, ^6^, ^7^, ^8^ In people and in other veterinary species, other acute phase proteins (APP) are used as important diagnostic tools to detect early inflammation and infection.^9^ Acute phase proteins are blood proteins that play an important role in the host's innate immune defences. They increase in concentration in response to external and internal challenges, such as inflammation, infection, and injury. To improve the early diagnosis of pneumonia in foals, it is essential to identify reliable APP biomarkers.

C‐reactive protein (CRP) is a positive APP that is produced in the liver and mainly regulated by proinflammatory cytokines, such as interleukin‐6 (IL‐6).^10^ In many species, CRP is a major APP that increases 10 to 1000‐fold in response to many infectious diseases.^11^, ^12^ In contrast, in horses, CRP is considered a moderate APP that increases within 3–5 days after an inflammatory event, but to a lower degree than in other species.^9^, ^13^ There are only a few studies documenting modest increases in CRP in horses with inflammatory conditions such as pneumonia, enteritis, or laminitis. In foals, there is a small body of literature on CRP concentrations after an inflammatory event. Age‐related changes in serum CRP concentration in foals have been documented, with very low concentrations at birth which then increase very rapidly and are dependent on colostrum intake.^13^ Another study that examined CRP concentrations in critically ill foals reported an increase in foals with inflammatory conditions like enterocolitis, colic, and septic arthritis, but CRP increases were not indicative of neonatal sepsis or associated with survival.^14^

In addition to the role it plays in regulating CRP, IL‐6 has also been identified as a potential biomarker in horses with enterocolitis, exercise‐induced acute‐phase response, and equine gastric ulcer syndrome.^15^, ^16^, ^17^ There are conflicting reports about changes in IL‐6 gene expression in cells from septic foals. In one report, gene expression of IL‐6 was higher in non‐surviving septic foals but lower overall compared to age‐matched healthy controls, and in another study, IL‐6 expression did not differ between septic and healthy foals.^18^, ^19^ Exogenous IL‐6 exposure has also been shown to increase the bactericidal activity of equine monocyte‐derived macrophages in an in vitro model of *R. equi* infection, suggesting this cytokine may play an important role in foal pneumonia.^20^ However, there are currently no reports measuring circulating IL‐6 concentrations in foals with naturally occurring bronchopneumonia.

In human medicine, both CRP and IL‐6 are important biomarkers in the diagnosis of neonatal infections, including sepsis and pneumonia.^12^, ^21^ However, the combination of plasma CRP and IL‐6 has not been evaluated as a predictor of respiratory disease in foals naturally exposed to *R. equi* and *Strep. zoo*. on endemic farms. The aim of this study was to evaluate plasma CRP and IL‐6 concentrations as potential biomarkers for early diagnosis of pneumonia in foals. Specifically, we measured CRP and IL‐6 concentrations at multiple time points from birth to weaning in foals on a farm with endemic foal pneumonia and evaluated associations between these markers and the development of respiratory disease in foals during this period. We hypothesised that both CRP and IL‐6 concentrations would be highest during the neonatal period and also in foals diagnosed with pneumonia compared to healthy age‐matched foals.

## 2 MATERIALS AND METHODS

The study was a prospective cohort study conducted at a large warmblood stud farm in Germany. The stud has a history of endemic foal pneumonia, primarily due to *R. equi* and/or *Strep. zoo*. infection.^22^, ^23^, ^24^ The included foals were born on the farm during the 2021 and 2022 breeding seasons and remained there until weaning.

### 2.1 General monitoring of the foals

All foals at the stud were examined at least once weekly from birth until the age of 5.5 months, when they are separated from their mares at weaning. The examination included the measurement of body temperature, evaluation of mandibular lymph nodes as well as nasal discharge, auscultation of the trachea and lungs, and trans‐thoracic ultrasonography in all foals, and measurement of white blood cell count (WBC) in foals with mild to severe bronchopneumonia (ultrasonographic abscess score >15 cm).^24^ For the ultrasound examination, portable equipment was used with a 7.5 MHz linear transducer, and both sides of the thorax from the 3rd to the 16th intercostal space were scanned from dorsal to ventral. As previously described, a pulmonary abscess was defined as a focal area of pulmonary consolidation with a diameter ≥ 1 cm.^8^, ^24^ The largest diameter of each consolidated region was measured and added together to generate the final abscess score.^8^, ^24^

### 2.2 Study design and inclusion criteria

The study included 200 initially healthy warmblood foals, with 100 foals sampled in each breeding season. The foals were born in clean and disinfected foaling boxes and spent their first week of life with their mothers in a separate stable. After this period, they were kept in small mare/foal groups in covered open stalls. From approximately 1 month of age, they were housed together on pasture in groups of 20 to 25 mare/foal pairs until they were weaned at the age of 5.5 months. Foals were included in the study if they had an uncomplicated, full‐term (≥ 330 days gestation) parturition, a normal clinical exam after birth, and documentation of appropriate transfer of maternal antibodies (IgG‐level ≥800 mg/dL via capillary electrophoresis) on Day 1 of life.

Seven blood samples were collected from each foal in the study from birth to 20 weeks of age as part of weekly health examinations. Specifically, blood samples were collected from the jugular vein into EDTA tubes within 12–24 h of birth, and at weeks 1, 2, 4, 8, 12, and 20 of life. Tubes were centrifuged (400 g for 20 min), and plasma was removed and transferred to 1.5 mL Eppendorf tubes for storage at −80°C. Samples were shipped on dry ice by a commercial transportation company (World Courier) to the University of Georgia for later batch analysis.

After weaning, the 200 initially healthy foals were divided into three health groups on the basis of the clinical and ultrasonographical findings collected at weekly health assessments described above. From each group, 30 foals were randomly chosen for downstream analysis of plasma samples (see next section for sample size justification). The foals with no clinical signs and an abscess score ≤ 1 cm at every exam until weaning were assigned to the healthy group (healthy). The subclinical group (subclinical) included foals with only small pulmonary abscesses (abscess score <15 cm), rectal temperature ≤39°C, and no or only mild clinical signs (such as mildly abnormal respiratory sounds) that resolved without antimicrobial treatment. The clinical group (clinical) included foals with large abscesses (abscess score ≥ 15 cm) and/or clinical signs of pneumonia such as fever, abnormal respiratory sounds, nasal discharge, and/or increased WBC (>13,000 cells/μL). Foals in the clinical group were treated based on the routine antimicrobial treatment protocol for foals on this farm with moderate to severe bronchopneumonia, with rifampin (10 mg/kg PO once daily) in combination with tulathromycin (2.5 mg/kg IM or IV once in a week). The treatment was carried out for at least 3 weeks, and there was a weekly rotation of IM and IV tulathromycin administration during the treatment.^25^ Foals with fever (>39.0°C) were also initially treated with metamizole sodium (30 mg/kg twice daily IV) as an anti‐pyretic.

### 2.3 Plasma C reactive protein and interleukin‐6 analysis

Thirty foals from each health group described above were randomly selected (https://www.randomizer.org) for sample downstream analysis of CRP and IL‐6 (*n* = 90 total). This number is based on sample size analysis using published IL‐6 data in septic and healthy foals at birth that revealed 30 foals per group would allow us to detect an approximately 6‐fold difference in IL‐6 concentrations between groups as significantly different, with α set at 0.5 and statistical power set at 80% (https://www.biomath.info/power/ttest.htm).^26^

In addition, a subset of foals (*n* = 20/group) were age‐matched for a direct comparison of IL‐6 and CRP concentrations in healthy and sick foals on the same day of life and at the timepoint closest to the time of subclinical (abscess score >1 < 15 cm) or clinical (clinical signs of pneumonia and/or abscess score ≥15 cm) diagnosis. Sample size for age‐matching was limited by the number of foals in the healthy group, as fewer foals remained healthy through 20 weeks of age (36/200) compared to the number of foals in the other two groups (subclinical 70/200 and clinical 93/200, with one foal's health status undetermined due to untimely accidental death) and also by the ability to align birth dates.

### 2.4 Laboratory methods

#### 2.4.1 Interleukin‐6

EDTA plasma was analysed for the presence of interleukin (IL)‐6 using a commercial enzyme‐linked immunosorbent assay (ELISA) kit (R&D Duoset) according to manufacturer directions, as previously validated and described.^26^, ^27^, ^28^ Reported lower and upper limits of detection were 125–8000 pg/mL. Assays were validated for equine plasma, and inter/intra coefficients of variation were calculated to be 6.4% and 13.7%, respectively, and R‐squared value of 0.99. Assay samples above the standard curve were diluted as necessary to fall within the linear portion of the standard curve. A four‐parameter logistic equation was created using GraphPad Prism version 10.0 (Graphpad Software LLC) to interpolate the concentration of each sample against the standard curve.

#### 2.4.2 C‐reactive protein

CRP was measured in EDTA plasma using a commercially available enzyme‐linked immunosorbent assay (ELISA) (ABCAM) according to manufacturer directions. Briefly, all kit reagents were equilibrated to room temperature prior to usage. Serial dilutions of the provided recombinant standard were performed to prepare a 6‐point standard curve. Samples were diluted 1:100–1:400 to fit within the standard curve. Reported lower and upper limits of detection were 6.25–200 ng/mL. Assays were validated for equine plasma and inter/intra coefficients of variation were calculated to be 5.0% and 11.0%, respectively, with an R‐squared value of 0.99. Samples above the standard curve were diluted as necessary to fall within the linear portion of the standard curve. A four‐parameter logistic equation was created using GraphPad Prism version 10.0 (Graphpad Software LLC) to interpolate the concentration of each sample against the standard curve.

### 2.5 Data analysis

Normality of the data and equality of variances were assessed using the Shapiro–Wilk and Levene's tests, respectively. Variables that did not meet the assumptions for parametric testing were log‐transformed prior to analysis. IL‐6 and CRP concentrations were compared among foal health groups and ages using linear mixed models. Age and health were modelled as fixed nominal effects, and foal was included as a random effect to account for repeated measurements within the same animals. Pairwise comparisons were performed using the Bonferroni procedure to limit the type I error probability to 5% over all comparisons. Analyses were performed using commercially available statistical software (Stata version 18.0, StataCorp LLC). Age‐matched comparisons were conducted with the Wilcoxon matched‐pairs signed rank test. All tests assumed a two‐sided alternative hypothesis, and values of *p* < 0.05 were considered statistically significant. Analyses were performed using commercially available statistical software GraphPad Prism version 10.2.0 (Graphpad Software LLC).

## 3 RESULTS

### 3.1 Clinical data

Clinical data and median (range) age of foals in each of the three health groups (*n* = 30/group) on the day of respiratory disease diagnosis (if relevant) are summarised in Table 1. All foals in the healthy group (*n* = 30) remained afebrile with no clinical signs of respiratory disease or pulmonary lesions on weekly trans‐thoracic ultrasonography at all timepoints from birth to weaning. In the subclinical group, only 5/30 foals had a rectal temperature >39°C, none showed overt clinical signs of respiratory disease, and all foals had only small pulmonary abscesses on trans‐thoracic ultrasonography (total abscess score <5 cm in 28/30 foals and 6–14 cm in 2/30 foals). In contrast, in the clinical group, 17/30 foals were febrile (rectal temperature >39°C) and all had a pulmonary abscess score above 10 cm (total abscess score 6–14 cm in 2/30 foals and >15 cm in 28/30 foals). The two clinical foals with a smaller total pulmonary abscess score had overt clinical signs of respiratory disease. In addition, 21/30 clinical foals had a WBC >13.0 × 10^9^/L on the day of respiratory disease diagnosis. All foals in the clinical group were placed on antimicrobial treatment according to the stud's treatment protocol as detailed above, starting on the day of clinical respiratory disease diagnosis. All 90 foals in the study population in which CRP and IL‐6 were measured survived to weaning. Of the initial healthy 200 enrolled foals that were used to establish the 30 foals/health group in which CRP and IL‐6 were measured, a total of five foals died from causes unrelated to respiratory disease during the sampling period (2021: *n* = 1; 2022: *n* = 4). The samples of these five foals were not analysed for CRP and IL‐6.

**TABLE 1:** Clinical parameters of 30 foals randomly classified into three health categories and used for quantification of plasma IL‐6 and CRP concentrations in this study.

Physical examination parameters	Healthy (*n* = 30)	Subclinical (*n* = 30)	Clinical (*n* = 30)
Temperature < 39.0°C	30	25	13
Temperature > 39.0°C	0	5	17
Abscess Score 1–5 cm	0	28	0
Abscess Score 6–14 cm	0	2	2
Abscess Score > 15 cm	0	0	28
WBC > 13.0 × 10^9^/L	Not measured	Not measured	21
WBC < 13.0 × 10^9^/L	Not measured	Not measured	9
Age (in weeks) at diagnosis [Mean, median (range)]	N/A	13.3, 13.6 (3.5–19.7)	13.7, 15.4 (7.2–19.7)

### 3.2 C reactive protein

**FIGURE 1:**
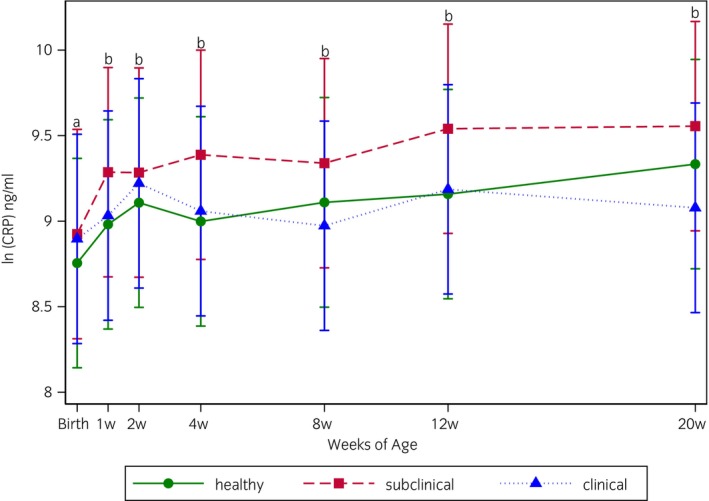
The natural log of the mean plasma concentrations (ng/mL) of CRP in 90 warmblood foals on a farm with endemic foal pneumonia at birth and at 1, 2, 4, 8, 12, and 20 weeks of life as measured by ELISA. The mean and 95% confidence interval of CRP measured in plasma of foals divided by respiratory health status as determined by weekly clinical exam and trans‐thoracic ultrasonography are represented by symbols and vertical lines (green circle (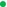) = healthy; red square (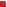) = subclinical pulmonary lesions; blue triangle (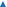) = clinical pneumonia). Foals in the healthy group remained healthy from birth to weaning, and foals in the subclinical and clinical groups had respiratory disease evident at least one weekly exam during this time period. An overall significant effect of age was detected in all groups (*p* < 0.001), but no significant differences were detected between foals of different health status (*p* = 0.77). Timepoint marginal means with letters in common do not differ with a level of significance of 5% for overall comparisons. Significance was set at *p* < 0.05.

**FIGURE 2:**
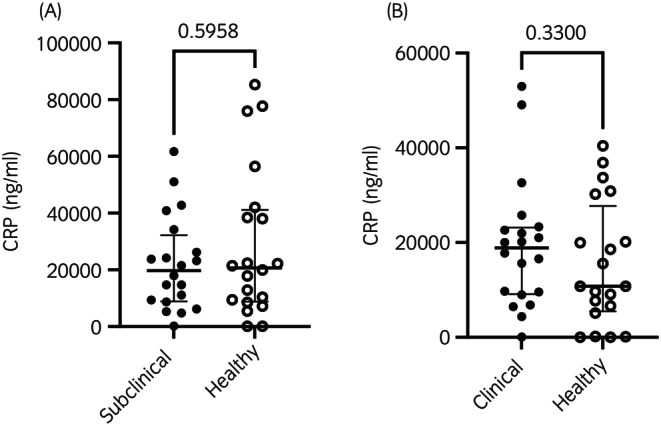
The median and interquartile range of plasma concentration of CRP (ng/mL) measured by ELISA in healthy foals (*n* = 20) and foals with subclinical pulmonary lesions (*n* = 20) or clinical pneumonia (*n* = 20). Foals in the subclinical and clinical groups were matched with healthy foals by age at the time of respiratory disease diagnosis. (A): Healthy foals compared to subclinical foals. Horizontal lines represent the median in each group, and individual foal measurements are represented by symbols (A) ● = subclinical; ○ = healthy and (B) ● = clinical; ○ = healthy. No significant (*p* < 0.05) differences between age‐matched controls were detected.

Plasma CRP concentrations in foals among health groups from birth to 20 weeks of age are shown in Figure 1. In a factorial analysis, there was no significant main effect of health status (*p* = 0.8), but there was a significant main effect of age (*p* < 0.001), with the mean ln(CRP) concentrations from Week 1 through Week 20 all being significantly higher than the mean ln(CRP) concentration at birth. There were no significant differences in the mean ln(CRP) concentrations from Week 1 through Week 20. There was no significant interaction between the effects of age and health status (*p* = 0.3). Individual variation among foals for all time points is shown in Figure S1A. In age‐matched foals, there was no significant difference between CRP plasma concentrations in subclinical (median 19,737 ng/mL, interquartile range 8902–32,241 ng/mL) and healthy (median 20,716 ng/mL, interquartile range 8793–41,119 ng/mL) (*p* = 0.6) (Figure 2A) or between the clinical (median 18,899 ng/mL, interquartile range 9121–23,159 ng/mL) and healthy (median 10,768 ng/mL, interquartile range 5498–27,724 ng/mL) groups (*p* = 0.3) (Figure 2B).

### 3.3 Interleukin‐6

**FIGURE 3:**
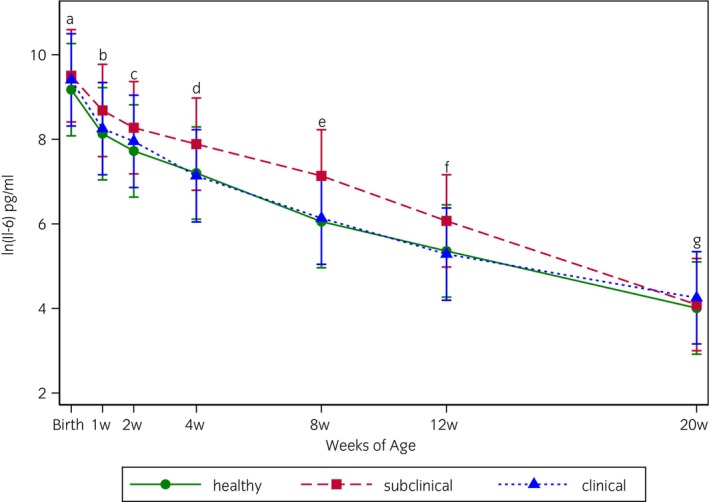
The natural log of the mean concentration of IL‐6 (pg/mL) from plasma of 90 warmblood foals on a farm with endemic foal pneumonia at birth and at 1, 2, 4, 8, 12 and 20 weeks of life as measured by ELISA. The mean and 95% confidence interval for foals by health status are represented by vertical lines and symbols (green circle (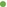) = healthy; red square (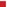) = subclinical; blue triangle (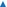) = clinical). Foals in the healthy group remained healthy from birth to weaning, and foals in the subclinical and clinical groups had respiratory disease evident at least one weekly exam during this time period. An overall effect of age was detected in all groups (*p* < 0.001), but no significant differences were detected between foals of different health status (*p* = 0.69). Timepoint marginal means with letters in common do not differ with a level of significance of 5% for overall comparisons. Significance was set at *p* < 0.05.

**FIGURE 4:**
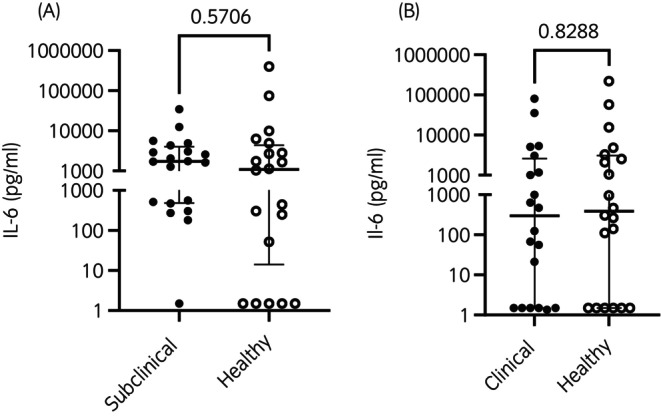
The median and interquartile range of plasma concentrations of IL‐6 (ng/mL) in age‐matched foals as measured by ELISA in healthy foals (*n* = 20) and foals with subclinical pulmonary lesions (*n* = 20) or clinical pneumonia (*n* = 20). Foals in the subclinical and clinical groups were matched with healthy foals by age at the time of respiratory disease diagnosis. (A): Healthy foals compared to subclinical foals. Horizontal bars represent median and individual foal measurements are represented by symbols ● = subclinical; ○ = healthy. (B): Healthy foals compared to clinical foals. Horizontal bars represent median and individual foal measurements are represented by symbols ● = subclinical; ○ = healthy. No significant (*p* < 0.05) differences between age‐matched controls were detected.

IL‐6 concentrations in healthy, subclinical, and clinical foals from birth to 20 weeks of age are shown in Figure 3. In a factorial analysis, there was no significant main effect of health status (*p* = 0.7), but there was a significant main effect of age (*p* < 0.001), with the mean ln(IL‐6) concentration being highest at birth and decreasing consistently through Week 20. Mean ln(IL‐6) concentrations of all weeks were significantly different from one another. There was no significant interaction between the effects of age and health status (*p* = 0.9). Individual variation among foals for all time points is shown in Figure S1B. IL‐6 concentrations were not different between age‐matched foals in the subclinical (median 1725 pg/mL, interquartile range 482–3987 pg/mL) and healthy group (median 1078 pg/mL, interquartile range 14–4370 pg/mL) (*p* = 0.6) (Figure 4A) or the clinical (median 297 pg/mL, interquartile range 1.5–2579 pg/mL) and healthy group (median 384, interquartile range 1.5–3099 pg/mL) (*p* = 0.8) (Figure 4B).

## 4 DISCUSSION

Identifying reliable early predictors for bronchopneumonia could decrease morbidity and mortality, especially on farms endemic for disease. In previous studies, IL‐6 and CRP concentrations have been suggested as good candidates for biomarkers during bacterial infections in the horse.^13^, ^16^, ^26^ This study is the first to evaluate plasma concentrations of CRP and IL‐6 during naturally occurring bronchopneumonia in foals. Our findings support our first hypothesis, showing age‐related changes in plasma CRP and IL‐6 concentrations from birth to weaning in foals, regardless of health status. Our data revealed an increase in CRP values from birth to Week 1, with no significant change from Week 1 to Week 20, consistent with a previous report that showed increasing CRP concentrations in healthy foals from birth to 1 year of age.^13^ Exposure to minor ex‐utero physical trauma or post‐parturient colonisation with normal microflora or exposure to other infectious organisms after birth may result in this increase in CRP concentrations in foals. Other studies also indicated that prior to colostrum intake, newborn foals have low or undetectable concentrations of plasma CRP.^13^, ^14^ It has been proposed that colostrum intake itself might directly provoke this post‐parturient elevation of CRP, as it is also described in calves.^13^, ^14^, ^29^ The foals in our study had measurable concentrations of CRP in our initial sample, but it is important to note that our initial birth sample was taken at approximately 12–24 h of age, after colostrum intake in all foals. Further study to quantify CRP in equine colostrum and determine the specific influence of colostrum intake on CRP production in foals is warranted.

Our data did not support our second hypothesis, as we did not detect health‐associated changes in plasma CRP or IL‐6 concentrations at any age or specifically in samples most closely aligned with the time of the initial respiratory disease diagnosis. Other reports have shown an increase in CRP in horses as a result of bacterial infection.^13^, ^30^ In contrast to our findings, Yamashita et al. compared CRP concentrations in foals (ages 3 days to 12 months) with pneumonitis (cause unspecified) to healthy adult horses and found a moderate but significant increase in concentrations, leading them to conclude that CRP might be a useful biomarker during acute inflammatory stimulation in foals.^13^ Another study reported that plasma CRP was increased in foals with non‐specific inflammation (enterocolitis, rib fractures, septic arthritis), but did not differentiate between septic and sick non‐septic foals, and recommended repeated measurements to increase predictive usefulness.^14^ Horses are considered moderate responders for CRP, as CRP levels only increase up to 10‐fold in horses during an inflammatory event.^13^, ^30^ In contrast, in dogs, which are classified as a major responder, CRP levels increase up to 100‐fold in an inflammatory event compared to low basal levels in healthy individuals.^9^, ^11^ Significant increases in CRP concentration were found in dogs at the time of bronchopneumonia diagnosis,^31^ It is possible that our failure to detect an increase in CRP associated with respiratory disease in foals in this study reflects an age‐ or species‐related difference in foals or might be associated with differences in the timing of sample collection among these studies. While foals in our study were monitored weekly, blood samples for CRP and IL‐6 quantification were only taken every 4 weeks after foals were 1 month of age, so the closest sample could have been up to 3 weeks after the initial disease diagnosis. CRP has a half‐life of 19 h, and peak values are reached 3–5 days post inflammatory stimuli,^10^, ^13^ Therefore, it is likely the infrequent timing of our later samples was insufficient to detect acute, infection‐related changes in CRP concentrations. Future sampling at the time of diagnosis and initial onset of disease and treatment, and daily for several days post diagnosis would be helpful to determine if there is diagnostic or prognostic value in measuring CRP in these sick foals.

IL‐6 is a proinflammatory cytokine which, among other effects, up‐regulates synthesis of acute phase proteins like CRP, fibrinogen, and serum amyloid A.^19^, ^32^ Il‐6 is predominantly produced by leukocytes in response to inflammatory stimuli or trauma.^26^ Our data demonstrated a significant age effect on IL‐6 concentration in foals, with the highest concentrations found shortly after birth. One reason for this finding may be that there is substantial transfer of IL‐6 in the colostrum, as equine colostrum, like in other animals, contains IL‐6.^26^, ^33^ The foals in the current study were all sampled after initial colostral intake, and all were initially healthy and with adequate transfer of maternal antibodies (≥800 mg/dL). Therefore, it is possible that the initially higher levels of IL‐6 reflect maternally‐derived IL‐6 from colostrum, and the decrease over time represents degradation and decreased production of IL‐6 as the foals aged.

There are no reports of plasma IL‐6 concentrations of foals with bronchopneumonia, either from an experimental infection model or in naturally occurring disease. Laboratory studies have shown that bronchoalveolar macrophages cultured ex vivo with *R. equi* produced IL‐6, most significantly after foals had been treated with a host‐directed therapeutic.^20^ Furthermore, IL‐6 mRNA expression from foal neutrophils increases significantly after stimulation with *R. equi* in vitro, with the greatest expression in neutrophils from foals ≤ Day 1 of life compared to neutrophils from older foals.^34^ These in vitro infection models demonstrate that foal leukocytes are capable of producing IL‐6 in response to bacterial infections, and suggest that it might have the potential to serve as a useful measure of disease in foals with naturally occurring bronchopneumonia despite the lack of a significant association between IL‐6 concentrations and subclinical or clinical respiratory disease in foals in this study. It is possible that an increase in IL‐6 concentrations produced at the pulmonary tissue level is not reflected when measuring circulating concentrations in plasma. Again, the timing of our sample collection may also have inhibited our ability to detect acute and transient changes in circulating IL‐6 associated with the onset of respiratory disease. Future studies investigating quantification of this cytokine more frequently and at the time of respiratory disease diagnosis are needed.

As discussed above, the major limitation to our study was the timing of our sample collection. More frequent (weekly) blood collection in foals for the entire study duration would have allowed us to more closely align our assays with the time of diagnosis and possibly allowed us to detect transient disease‐associated changes in IL‐6 and CRP. Additionally, daily sample collection at and after the time of diagnosis would also be helpful to increase our understanding of how the availability of these proteins' changes in the initial stages. It should also be noted that the foals with fever in the subclinical and clinical groups were given an anti‐inflammatory drug (metamizole sodium) to reduce fever, which may well have led to a reduction in CRP and IL‐6 concentrations at subsequent time points. Bronchopneumonia diagnosis was made based on clinical signs and trans‐thoracic sonographic findings and was not confirmed with further diagnostic tools, such as thoracic radiography or cultures of transtracheal aspirate samples, which is an additional important limitation of our study.

In sum, on a farm with endemic foal bronchopneumonia, circulating plasma CRP and IL‐6 concentrations varied as foals aged from birth to weaning, but neither protein was associated with the development of respiratory disease during this period in foals. Further study with more frequent sampling in a larger number of foals is needed to determine if there is any diagnostic or prognostic value in measuring CRP or IL‐6 in foals at risk of bronchopneumonia.

## FUNDING INFORMATION

This work was supported by Paul Schockemoehle Pferdehaltung GmbH, Lewitz Stud, Neustadt‐Glewe, Germany and University of Georgia, College of Veterinary Medicine, Marguerite Thomas Hodgson Equine Studies Endowment.

## CONFLICT OF INTEREST STATEMENT

The authors declare no conflicts of interest.

## AUTHOR CONTRIBUTIONS

**Dorothea Hildebrandt:** Writing – original draft; data curation. **Monica Venner:** Supervision; writing – original draft; funding acquisition. **Kelsey A. Hart:** Writing – review and editing; funding acquisition; supervision. **Londa Berghaus:** Conceptualization; writing – original draft; data curation; formal analysis.

## DATA INTEGRITY STATEMENT

Kelsey A. Hart and Londa Berghaus had full access to the study data and take responsibility for the data integrity and analysis accuracy.

## ETHICAL ANIMAL RESEARCH

Study design and data collection was approved by the University of Georgia College of Veterinary Medicine Clinical Research Committee.

## INFORMED CONSENT

Owners gave consent for their animals' inclusion in the study.

## Supporting information


**Figure S1.** The measured concentration of CRP in ng/mL (A) and IL‐6 in pg/mL (B) from plasma of 90 warmblood foals on a farm with endemic foal pneumonia at birth and at 1, 2, 4, 8, 12, and 20 weeks of life as measured by ELISA. Foals were divided by health status, and the concentration of individual foals is represented by symbols for each health group (● = healthy; ■ = subclinical; ▲ = clinical). Data are presented to graphically demonstrate the individual variation of concentrations of measured parameters among foals and is for descriptive purposes only.

## Data Availability

The data that support the findings of this study are openly available in *Mendelay Data*: Berghaus, Londa (2024), ‘C‐reactive Protein and Interleukin 6 in foals’, *Mendeley Data*, V1, doi: 10.17632/cthsph4r3g.1.
