# Supplementary figures and images for: Plasma C‐reactive protein and interleukin‐6 concentrations in foals during health and respiratory disease
Source: Equine Vet J. 2025 Jul 20;58(2):372–9. doi: 10.1111/evj.70000 (PMC12892378; doi:10.1111/evj.70000)

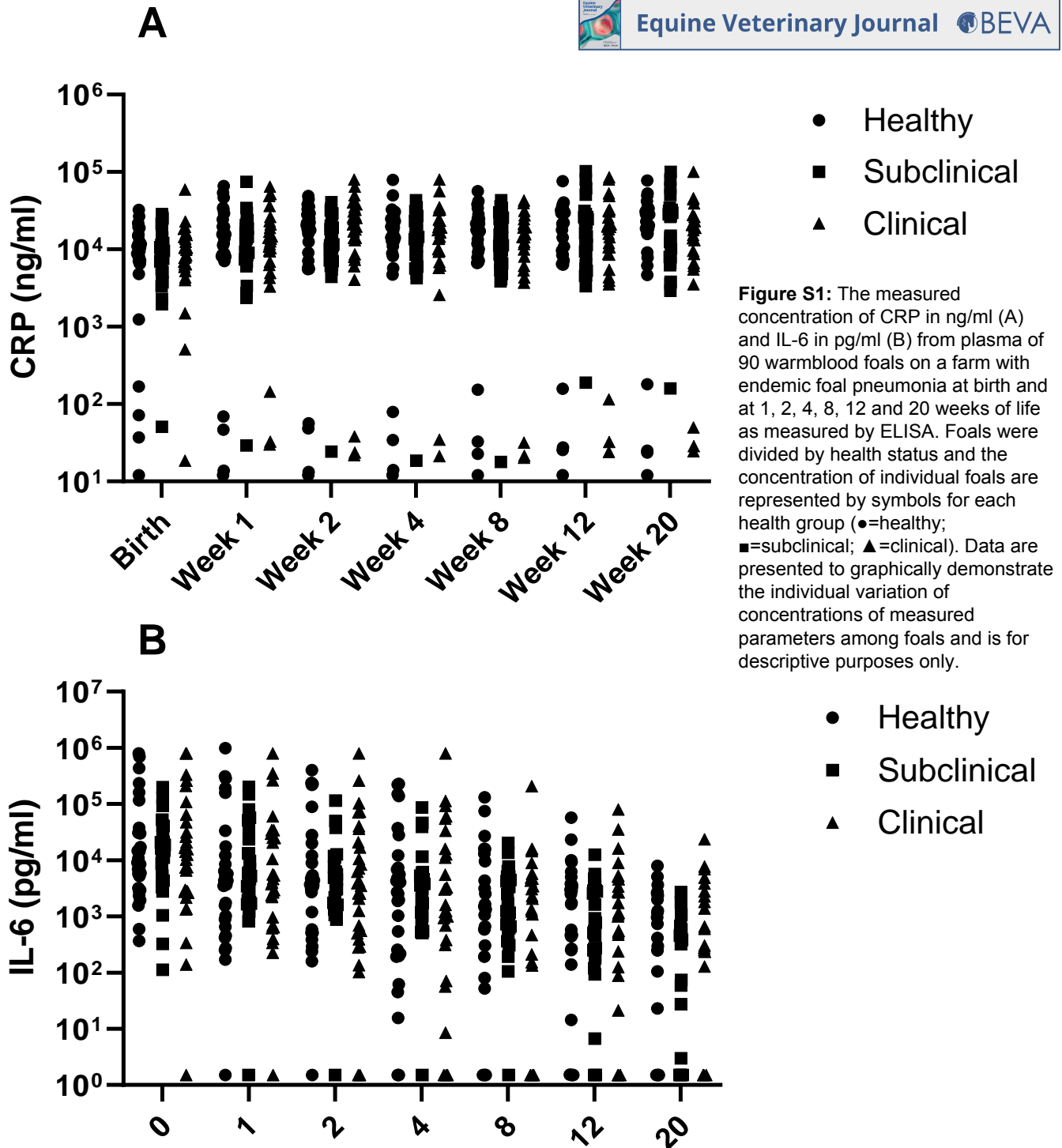

Supplement: Supplementary file 1 — Figure S1. The measured concentration of CRP in ng/mL (A) and IL‐6 in pg/mL (B) from plasma of 90 warmblood foals on a farm with endemic foal pneumonia at birth and at 1, 2, 4, 8, 12, and 20 weeks of life as measured by ELISA. Foals were divided by health status, and the concentration of individual foals is represented by symbols for each health group (● = healthy; ■ = subclinical; ▲ = clinical). Data are presented to graphically demonstrate the individual variation of concentrations of measured parameters among foals and is for descriptive purposes only. [file EVJ-58-372-s001.pdf]
